# P-516. Implementation and Uptake of Long-acting Injectable PrEP at Atlanta VA Healthcare System

**DOI:** 10.1093/ofid/ofae631.715

**Published:** 2025-01-29

**Authors:** Nadine M Harris, Kathryn E DeSilva, Roseylee Kuriakose, Stacy Sorlie, Sarah Reed, Nora T Oliver, Abeer Moanna, Andrew S Webster, Emily J Cartwright

**Affiliations:** Atlanta VA Medical Center/Emory University SOM, Decatur, GA; Atlanta VA Medical Center, Decatur, Georgia; Atlanta VA Healthcare System, Decatur, Georgia; Atlanta VA Healthcare System, Decatur, Georgia; Atlanta VA Healthcare System, Decatur, Georgia; Atlanta VA Medical Center, Decatur, Georgia; Atlanta VA Medical Center, Decatur, Georgia; Atlanta VA Medical Center, Decatur, Georgia; Emory University School of Medicine, Atlanta VA health care system, Decatur, Georgia

## Abstract

**Background:**

Long-acting injectable (LAI) pre-exposure prophylaxis (PrEP) is highly effective at preventing HIV and has the potential to expand PrEP access. Atlanta VA Healthcare System (AVAHCS) has one of the largest PrEP programs within the Veteran’s Health Administration (VHA). In mid-2023, we started a LAI PrEP program and quickly became the largest provider of LAI PrEP within VHA. We aim to share our experience building this program as well as describe uptake amongst patients

**Figure:**
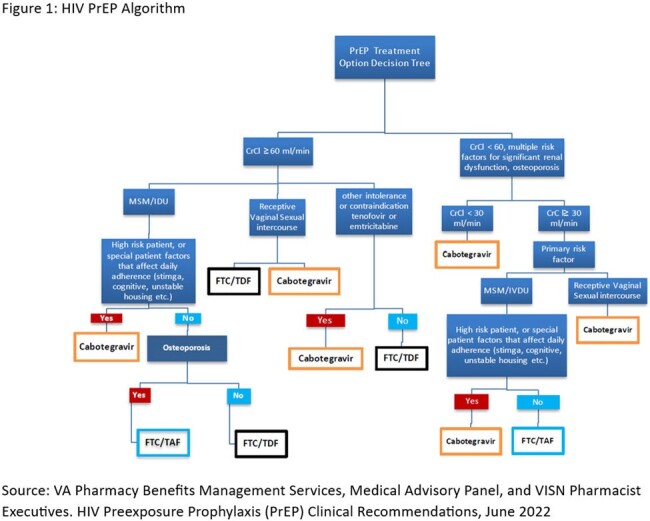

**Methods:**

In May of 2023, a dedicated advanced practice provider (APP) was hired to manage the LAI program, working closely with an Infectious Disease (ID) clinical pharmacist and ID nurse to refer patients for LAI PrEP based on VA criteria (Figure 1, Figure 2). Active LAI PrEP patients are added to a database tracker and reviewed at weekly interdisciplinary team rounds

**Figure:**
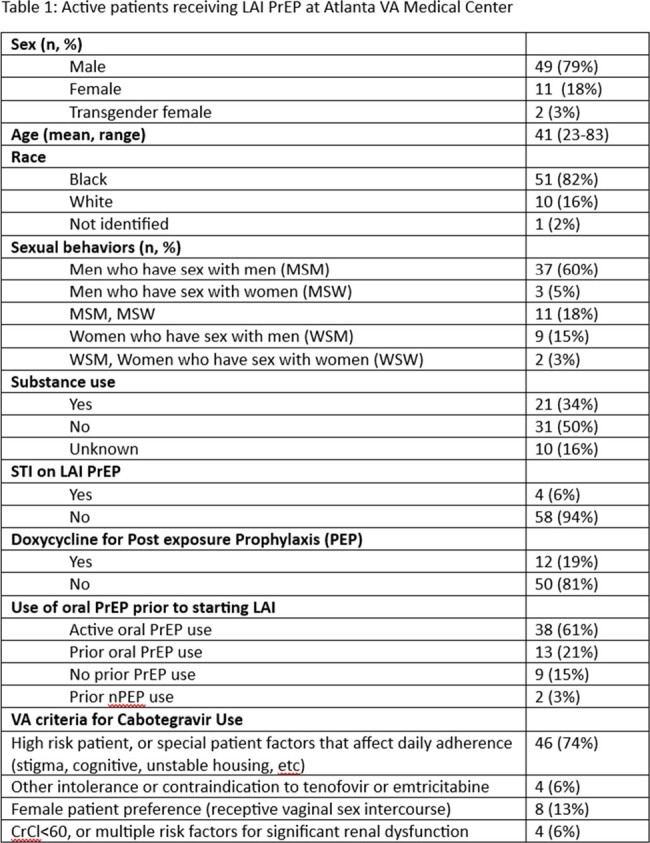

**Results:**

From May 2023 to April 2024, 77 patients were referred for LAI PrEP. There are 62 active LAI PrEP patients, with a mean age of 41 years (23-83 years). Most were men (n=49, 79%) and self-identified as black race (n=51, 82%). The most common indication for LAI PrEP use (n=46, 74%) was being at high risk of HIV acquisition, or having special patient factors that affect adherence to oral PrEP (Table 1). Of the 15 patients who were referred for LAI PrEP but discontinued use , reasons cited included: adverse reactions (n=4, 40%), inability to adhere to clinic scheduling (n=3, 30%), no longer desiring PrEP (n=2, 20%), and relocation to another state (n=1, 10%); 5 never initiated LAI PrEP. There were 7 patients (46%) who resumed oral PrEP after LAI discontinuation.

**Figure:**
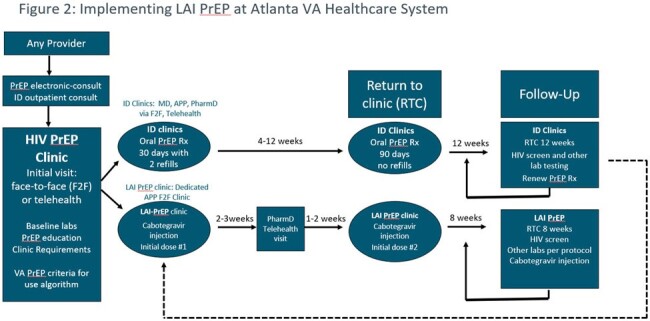

**Conclusion:**

We have rapidly expanded access to LAI PrEP at the AVAHCS using a model that may be replicated at other VA facilities. Assessment of risk and patient preference often guides the decision about starting LAI PrEP in our cohort and should be an important consideration when creating guidance about eligibility for LAI PrEP.

**Disclosures:**

**All Authors**: No reported disclosures

